# Institutionalizing convergence education for medical artificial intelligence

**DOI:** 10.1007/s13534-025-00523-2

**Published:** 2025-11-01

**Authors:** Tae In Park, Jongmo Seo, Hyung-Jin Yoon, Kyu Eun Lee

**Affiliations:** 1 Department of Medicine, Seoul National University College of Medicine, Seoul, 03080 Republic of Korea; 2 Department of Electrical and Computer Engineering, Seoul National University College of Engineering, Seoul, 08826 Republic of Korea; 3 Medical Big Data Research Center, Seoul National University College of Medicine, Seoul, 03080 Republic of Korea; 4 Department of Surgery, Seoul National University College of Medicine, Seoul, 03080 Republic of Korea

**Keywords:** Medical AI education, Convergence curriculum, Modular program design, Transdisciplinary training, Institutional governance, Digital health policy alignment

## Abstract

As artificial intelligence (AI) becomes increasingly central to modern healthcare, medical education must move beyond passive knowledge transfer and adopt a system-wide approach to convergence training. This narrative review shares a 5-year case study from Seoul National University College of Medicine (SNU Medicine), which developed a comprehensive, multi-level model for integrating AI into medical education. Instead of relying on pilot programs or piecemeal curriculum updates, SNU Medicine established a governance-driven, modular framework that includes institutional infrastructure, interdisciplinary teaching strategies, cross-campus credit integration, and alignment with national digital health policies. Based on this long-term case, we propose four key design principles—modularity, transdisciplinary alignment, infrastructure-curriculum coupling, and policy embeddedness—as a framework for creating scalable and sustainable convergence education in medical AI. While rooted in Korea’s unique policy environment, this model provides transferable insights for medical institutions worldwide, particularly those operating within public or policy-constrained environments.

## Introduction: shifting from technological disruption to educational transformation

The merging of artificial intelligence (AI), data science, and digital health is no longer just a future idea but an active force transforming medical education today. As machine learning tools are applied in clinical environments—from AI-assisted diagnostics to generative language models for clinical documentation—the skills healthcare professionals need are rapidly changing [1, 2]. Physicians are now expected not only to interpret biological signals and make clinical decisions but also to understand how algorithmic systems process data, the biases they may inherit, and how these biases influence patient care [3, 4]. As a result, medical education must urgently transition from a knowledge-transfer model to one that develops adaptive, interdisciplinary problem solvers ready for a digital healthcare environment.

The COVID-19 pandemic has sped up this shift. The global crisis transformed digital health and remote diagnostics from simple conveniences into vital services. As telemedicine expanded rapidly and AI-supported triage systems were widely adopted, the necessity for digital literacy in medicine became obvious. Institutions worldwide have started testing new approaches, including data-driven decision-making, medical informatics boot camps, AI simulation platforms, and hybrid learning environments tailored for digital-native physicians [5–7].

Despite this momentum, however, global efforts to reform medical education remain inconsistent, often broken up across institutions or isolated by discipline. There is an increasing recognition that AI-related skills—such as computational thinking, data ethics, and interdisciplinary collaboration—cannot be taught as optional extras. They must be integrated into the fundamental structure of medical training [8–10]. Yet, implementing such change raises complex questions: Which models of integration are scalable? How can medical schools collaborate across engineering, data science, and clinical practice? How can convergence education prepare learners not only to use AI tools but also to influence their development and governance?

In this article, we examine these questions by analyzing the 5-year journey of Seoul National University College of Medicine (SNU Medicine) from September 2020 to August 2025. In doing so, we illustrate not only why convergence education is needed, but also how it can be implemented. Based on the SNU Medicine case, we propose four design principles—modularity, transdisciplinary alignment, infrastructure–curriculum coupling, and policy embeddedness—that serve as a framework for scalable medical AI education.

Unlike typical case reports, which often focus on pilot programs or isolated initiatives, this review presents the SNU Medicine model as a long-term institutional experiment in cross-disciplinary, AI-driven medical education. Using a 5-year timeline that includes program innovation, curricular redesign, government-academic collaboration, and global partnerships, we extract practical insights and policy-relevant implications that could guide similar efforts worldwide.

The goal is not to provide a blueprint to copy but to present a platform model that can be questioned: What is necessary to establish convergence education at the crossroads of medicine, engineering, and AI? What infrastructure, governance, and partnerships are needed? And how can such models stay flexible in a quickly changing technological environment?

## Why AI and convergence matter in medical education: revisiting the foundations

The concept of convergence education—combining various disciplinary perspectives to address complex, real-world problems—has gained renewed relevance in the AI era. In medicine, convergence refers to the integration of traditionally separate fields, including life sciences, engineering, computer science, behavioral science, and ethics. This is not just a pedagogical trend, but a fundamental necessity in complex clinical settings where data from wearable sensors, imaging platforms, genomic assays, and electronic health records (EHRs) inform multi-layered decision-making pipelines.

In the United States Government’s Interagency Working Group on Convergence 2022 report, *Convergence Education: A Guide to Transdisciplinary STEM Learning and Teaching*, it is described as being “driven by compelling or complex socio-scientific problems or topics, where learners apply knowledge and skills using a blended approach across multiple disciplines (i.e., transdisciplinary) to create and innovate new solutions” [11]. This characterization highlights both the problem-centered nature and the transdisciplinary scope of convergence education, making it particularly relevant to the challenges posed by AI in healthcare. In medicine, convergence refers to the integration of traditionally separate fields, including life sciences, engineering, computer science, behavioral science, and ethics [12–15]. This is not just a pedagogical trend, but a fundamental necessity in complex clinical settings where data from wearable sensors, imaging platforms, genomic assays, and electronic health records (EHRs) inform multi-layered decision-making pipelines.

Several medical and engineering schools worldwide are integrating AI and data science into biomedical education through interdisciplinary approaches. A well-known example is the Harvard–MIT Health Sciences and Technology (HST) program (hst.mit.edu), which has long served as a model for interdisciplinary training across engineering, medicine, and biomedical sciences. In recent years, it has increasingly focused on computational and AI-driven methods. At Imperial College London, students can learn AI and data science through specialized courses and programs, such as Health Data Analytics and Machine Learning, a Master’s course offered by the School of Public Health that equips learners with advanced competencies in analyzing and integrating complex health data sets using statistical and machine learning methods (imperial.ac.uk/study/courses/postgraduate-taught/health-data-analytics). Meanwhile, Georgia Tech College of Engineering’s joint Biomedical Engineering department with Emory University School of Medicine incorporates AI into its biomedical systems modeling and informatics courses, emphasizing data-driven techniques in core classes (bme.gatech.edu/bme). While these programs show practical ways to include AI literacy in biomedical education, they often operate in resource-rich, research-focused environments. However, their models may be hard to replicate in public medical schools or national systems with limited resources and different policy conditions.

What sets convergence education in medical AI apart is the need for a translational mindset. Students must not only understand algorithmic systems but also be capable of placing them within patient-centered care, population health objectives, and regulatory environments. This requires a shift from teaching “how to use tools” to training professionals who can co-design, evaluate, and govern those tools. Thus, convergence education must go beyond interdisciplinary exposure—it must develop new professional identities that bridge disciplines without becoming trapped by any one of them [16–18].

Furthermore, progress in medical education requires more than just curriculum reform. Institutional collaboration, faculty development, governance frameworks, and external partnerships must support it. It must also be modular and flexible—capable of being scaled up nationally or adapted for resource-limited environments [19–22]. In short, convergence education is both a policy and institutional initiative as well as a curricular effort.

In this context, the SNU Medicine case provides an opportunity to examine how a public medical institution within an East Asian policy environment has attempted to develop a convergence model—not as a single pathway, but as an interconnected educational ecosystem involving degree programs, microcredentials, national funding, international collaboration, and cross-campus governance. The following sections examine this case in three stages, analyze its key design components, and draw lessons for the worldwide biomedical engineering and medical education communities.

## Institutional trajectory (2020–2025): case phases

### Foundations (2020–2022)

SNU Medicine launched its convergence education strategy amid a time of significant global uncertainty and rapid technological advancement. Between 2020 and 2022, instead of launching isolated AI-themed lectures or elective seminars, SNU Medicine strategically laid the foundation for long-term change by focusing on three main areas: curricular experimentation, research infrastructure development, and cross-campus academic integration.

#### Curricular innovation via industry collaboration

A significant milestone in this phase was the August 2021 launch of the elective course “Deep Learning for Healthcare”, which was co-developed and co-taught with NAVER, Korea’s leading technology company. Instead of offering a typical AI survey course, this class was developed as an 8-week hybrid intensive program tailored explicitly for fourth-year medical students. It combined lectures on convolutional neural networks, recurrent architectures, and generative models with coding labs based on real clinical data, such as biosignals and EHRs. What made this initiative unique was the direct involvement of a NAVER AI research scientist, officially appointed as an adjunct professor, who co-taught the course with faculty from SNU Medicine. This university–industry collaboration model enabled students to investigate AI methods in a clinically relevant setting while gaining experience with real-world datasets and practical algorithmic thinking.

Student feedback from the initial course offering showed strong support, with over 85% highlighting the interdisciplinary format and practical relevance. However, feedback also pointed out challenges for students with limited programming experience, leading to adjustments in teaching methods. Although it was just one course, this partnership served as a testing ground for broader integration, demonstrating that industry collaborations could enhance convergence education without sacrificing academic standards.

#### Establishing a research infrastructure: the medical big data research center

A major element of the SNU Medicine’s early convergence initiatives was the establishment of the Medical Big Data Research Center (MBRC) in September 2021. Unlike traditional research institutes, MBRC was designed as an institutional platform to support both technological development and interdisciplinary talent development within Korea’s emerging digital health agenda. Funded through the national Information Technology Research Center (ITRC) program, MBRC focused on creating analytic frameworks for unstructured medical data—such as surgical videos, biosignals, and clinical audio—and on developing professionals capable of working across clinical, engineering, and data science fields. ITRC is a research and development support program aimed at universities, initiated by Korea’s Ministry of Science and ICT in 2000. It promotes research skills in emerging areas like AI, robotics, and data science. By July 2024, it has trained more than 17,800 graduate students.

In educational terms, the center played a dual role. First, it offered physical and computational infrastructure that allowed the integration of real-world datasets into student training. Graduate courses in medical data science, for example, were aligned with ongoing MBRC research projects, and student teams regularly participated in applied challenges such as clinical datathons and AI competitions. Second, MBRC assisted in prototyping a model for integrating AI education within a translational research setting—providing early insights into designing project-based, modular, and industry-connected learning experiences.

MBRC’s institutional positioning also mirrored broader policy shifts. As Korea’s Digital New Deal, which was launched in 2020 as its leading national strategy to boost digital transformation and innovation across essential sectors, focused on AI-driven healthcare innovation, MBRC emerged as one of the early institutional platforms aimed at integrating education, clinical research, and public investment. However, the model also exposed tensions every day in many convergence initiatives, including balancing research productivity with pedagogical goals, maintaining faculty engagement across disciplines, and meeting policy-driven performance metrics within tight deadlines. These dynamics underscore the challenge of achieving convergence through research centers alone and highlight the necessity of coordinating infrastructure development with curricular reform from the outset.

#### Cross-campus integration: the joint bachelor’s–master’s pathway

The third key initiative in this foundational phase was the launch of an integrated Bachelor’s–Master’s program in partnership with Seoul National University’s Graduate School of Data Science (GSDS). Launched in March 2022, this cross-campus pathway enables selected undergraduate medical students to initiate formal research in data science during their MD coursework and transition smoothly into a complete MS program upon graduation. Up to 30% of each GSDS master’s cohort can be admitted via this early admissions track. Students enrolled in the program participate in supervised research, gaining practical experience with biomedical data and building interdisciplinary skills that connect clinical medicine and computational analysis.

This initiative addressed two ongoing gaps in AI-medicine education: first, the lack of structured pathways for medical students to obtain formal qualifications in data science; and second, the gap between undergraduate clinical training and graduate-level computational education. By creating a scalable academic system that links multiple schools, SNU Medicine transitioned from sporadic interdisciplinary efforts to a more formalized structure for fostering convergence talent.

This program is further distinguished by its dual-campus governance and modular flexibility, allowing students to personalize their research focus with support from faculty in medicine and data science. According to official guidelines, participants must complete a designated set of research milestones and coursework in both schools, including a customized research project evaluated by a joint committee. This structure guarantees academic rigor while encouraging convergence-focused inquiry. In the context of convergence education, the program demonstrates how cross-school collaboration can break down traditional silos and promote integrated learning environments—an increasingly vital feature in training the next generation of biomedical AI professionals. This early experimentation also laid the foundation for modularity, one of the design principles later elaborated in Sect. 4.

### Institutionalization and expansion (2022–2024)

Between 2022 and 2024, SNU Medicine shifted from experimental initiatives to formal institutional consolidation. What initially began as pilot efforts developed into official programs, backed by new governance structures, dedicated academic tracks, and external collaborations. This period was characterized by two major developments: the creation of SNU AI.MED and the launch of structured curricula that incorporated AI into undergraduate and graduate medical education.

#### Creating an institutional platform: launch of SNU AI.MED

In July 2022, the SNU Medicine officially established the SNU Project Group for Education and Research in Medical AI (SNU AI.MED), a dedicated academic and strategic unit responsible for advancing the integration of AI into medical education. Its formation followed SNU Medicine’s selection for the Government Grant Program for Education and Research in Medical AI, a nationwide initiative jointly managed by the Ministry of Health and Welfare (MOHW) and the Ministry of Education (MOE), as they are Korea’s main government agencies in charge of healthcare and higher education policy. The grant program aims to promote integrated education and research in AI across medical, engineering, and data science fields, and to support institutional infrastructure capable of developing cross-disciplinary talent aligned with national digital health priorities.

Within this policy framework, SNU AI.MED was established not as a temporary project but as a sustainable organizational platform responsible for establishing convergence education. Its main responsibilities included curriculum development, coordination across departments, and program evaluation. Designed as both an academic working group and a governance mechanism, the unit served as a link between internal reform efforts and externally funded national strategies.

The mission of SNU AI.MED covered multiple areas, aiming to expand modular and interdisciplinary education at both undergraduate and graduate levels, promote translational research and industry collaboration, and develop credit-bearing, policy-aligned microcredentials. It successfully linked the College of Medicine with the College of Engineering, the Graduate School of Data Science, and university hospitals, establishing a university-wide framework for managing medical AI education.

As a result, the launch of SNU AI.MED marked a shift in SNU Medicine’s convergence strategy—from scattered, faculty-driven experiments to a university-backed, policy-connected approach to educational innovation. More than just a managerial response to external grant mechanisms, it signified a strategic shift in how medical education integrates into the broader digital transformation of healthcare, shaping governance, infrastructure, and long-term academic planning.

#### Expanding curricular offerings: pre-medical courses and microdegree programs

To support the development of early-stage talent, a new undergraduate course titled “Healthcare and Data Science” was launched in September 2023 as part of the pre-medical curriculum. The course covered vital topics in AI, medical data analysis, and data ethics, giving students their first structured introduction to computational thinking in a clinical setting. By teaching foundational skills before the clinical years, the course addressed a longstanding gap in medical training: the late inclusion of AI and data science instruction.

At the graduate level, SNU Medicine launched its Medical AI Microdegree Program in March 2024 as a postgraduate certification pathway. The Microdegree offered a formal, cross-listed curriculum drawing from medicine, data science, and bioengineering. The program included courses such as *Biomedical Signal Measurement*, *Genomic and Medical Data Analysis*, and *Ambient AI for the Medical Domain*. Importantly, this structure was aligned with SNU’s university-wide microdegree policy, emphasizing modularity, interdisciplinary recognition, and career relevance.

The microdegree program had three main goals: (1) certifying AI skills in various fields, (2) offering scalable models for integrated training, and (3) encouraging faculty from different departments to develop and teach courses together. This approach not only expanded the range of available courses but also promoted a culture of collaborative teaching.

#### Toward structured governance and evaluation

This period was marked not only by expanding the curriculum but also by strengthening institutional support. SNU AI.MED is guided by a steering committee made up of faculty from medicine, engineering, and data science, along with liaisons from university hospitals and industries. This ensured that program development responded to changing clinical and technological needs, while also aligning with national policy priorities related to digital health and workforce training.

At this stage, convergence education at SNU Medicine became more systematically organized—not just in its content but also in governance, evaluation, and funding. This institutional growth laid the groundwork for deeper global collaboration and formal recognition in the national digital health strategy. Together, these changes highlighted the importance of structured governance and cross-disciplinary collaboration, anticipating the design principle of transdisciplinary alignment elaborated in Sect. 4.

### Globalization and leap (2024–2025)

By 2024, SNU Medicine expanded its convergence efforts from merely institutional initiatives to a broader phase involving global partnerships, curriculum growth, and increased national recognition. This period marked a strategic leap, turning previously localized models—such as co-taught courses, microdegree programs, and cross-campus cooperation—into scalable frameworks for international collaboration and policy-linked education. Three key developments marked this phase: the Global Capstone Project (GCP), the launch of a joint major in Health Sciences and Technology (HST-K), and the university’s selection for a major national funding program in medical AI talent development.

#### Developing a global convergence model: the capstone project

In Fall 2024, SNU AI.MED launched GCP as a formal graduate-level course that merges education, research, and international mentorship. Building on earlier experimental exchanges, the course was created to enable students from medicine, engineering, and data science to form interdisciplinary teams and work on real-world AI healthcare projects under the guidance of global mentors. Partner institutions included the Massachusetts Institute of Technology, Harvard Medical School, Massachusetts General Hospital, National University of Singapore, and Pázmány Péter Catholic University.

The program employed a structured, end-to-end approach: students proposed project topics, paired with international mentors, completed global field training, and participated in a final presentation and peer review process. Instead of being a short-term international workshop, the capstone course was integrated into SNU’s academic credit system, providing formal recognition and academic rigor. GCP was not only an international educational initiative but also embedded within a national funding framework. It was developed as part of a government-supported program for global data convergence leadership in biomedicine, funded by Korea’s Ministry of Science and ICT (MSIT). This integration of international collaboration into a formal national policy scheme allowed the project to align global field-based learning with institutional and strategic goals in data governance, AI ethics, and translational research. Rather than being an isolated experiment, the course reflects a broader institutional shift toward incorporating international collaboration and mentor-based learning into formal convergence education frameworks.

In pedagogical terms, the capstone served multiple purposes: it integrated global standards into course design, offered exposure to diverse regulatory and healthcare environments, and provided iterative feedback from leading institutions. In institutional terms, it provided a reproducible model for hybrid, mentor-driven education that could be used in other convergence fields. More broadly, it reflected SNU Medicine’s strategic goal to make global collaboration a core part of its medical AI curriculum, not just an extracurricular or diplomatic act.

#### Toward a collaborative academic program: the launch of HST-K

Along with the globalization of the capstone, SNU Medicine began preparing to launch a new undergraduate joint major in Health Sciences and Technology (HST-K) in partnership with the College of Engineering. In late 2024, the two colleges established a joint planning committee and completed a university-funded policy research project to prepare for the program, which is tentatively scheduled to start in 2026. The curriculum was benchmarked against internationally recognized models, including the Harvard–MIT HST Program.

The HST-K major was created to fill longstanding gaps in the curriculum by incorporating convergence-centered fields—such as medical AI, biomedical engineering, regulatory science, and ethical innovation—into a formal undergraduate program jointly run by the College of Medicine and the College of Engineering. As a new intercollegiate program, its structure and governance are still being developed, reflecting both colleges’ common goal of fostering interdisciplinary talent at the undergraduate level.

Structurally, the HST-K initiative introduced a new level of institutional commitment to convergence education: it mandated credit integration across separate colleges, the creation of a joint admissions process, and cross-appointment of faculty for core curriculum delivery. From a governance standpoint, the program served as both a policy tool and an academic framework, aligning with national concerns about talent shortages in digital health and supporting the development of a new group of professionals capable of moving seamlessly across clinical, technological, and regulatory fields.

#### Achieving national recognition: talent development grant for 2025–2029

In April 2025, SNU Medicine was chosen as one of six institutions to receive a 5-year grant under the Korea Health Technology Research and Development (R&D) Project. This initiative, funded by MOHW, is managed by the Korea Health Industry Development Institute (KHIDI), a public organization under MOHW that oversees health technology R&D and supports the healthcare industry. The grant will support the continued growth of SNU AI.MED and its efforts to improve structured medical AI education. The program aims to train over 170 students, develop a comprehensive educational pipeline in AI from undergraduate to graduate levels, and expand modular microdegree programs along with integrated project-based learning opportunities.

Importantly, the program also encouraged university-wide collaboration. The funded consortium included not only the College of Medicine but also the College of Engineering, the School of Transdisciplinary Innovation, the Graduate School of Convergence Science and Technology, and university hospitals. Industry partners included NAVER Cloud, Kakao Healthcare, Microsoft, AWS, and others. Many global collaborators—initially engaged through the Capstone course—were formalized into long-term partnerships.

This institutional–national–global alignment marked a new phase in SNU Medicine’s strategy: convergence education was no longer an experimental reform or policy add-on, but a fully institutionalized, nationally funded, and globally networked area of strategic academic development. The groundwork laid between 2020 and 2024 has now created a platform capable of influencing both the structure and direction of medical AI education on a large scale. Such alignment illustrates the importance of policy embeddedness and infrastructure–curriculum coupling, principles elaborated in Sect. 4.

Overall, these three phases illustrate the development of SNU Medicine’s institutionalization of AI education. From this, several broader design principles emerge, providing a framework that goes beyond the specific context of SNU Medicine. Table 1 summarizes the phased development of convergence education from 2020 to 2025, aligning the three phases described above with their strategic focus areas and key milestones.

**Table 1:** Phased development of convergence education for medical AI at SNU medicine (2020–2025)

Phase	Strategic focus areas	Key initiatives and milestones
Phase 1: Foundations (2020–2022)	Industry collaboration on curriculum design Research infrastructure development Cross-campus academic collaboration	Introduction of the “Deep Learning for Healthcare” elective, co-taught with NAVER Establishment of MBRC with ITRC funding Initiation of BA–MS integrated pathway with GSDS
Phase 2: Institutionalization and Expansion (2022–2024)	Establishing a governance platform Implementation of a modular and interdisciplinary curriculum Engagement with national grant programs	Formation of SNU AI.MED through the MOHW–MOE national grant program Implementation of the pre-medical course “Healthcare and Data Science” Establishment of a Medical AI Microdegree Program Organization of a cross-departmental steering committee
Phase 3: Globalization and Leap (2024–2025)	Global project-based education and international mentoring Intercollegiate program planning Long-term national and international partnerships	Deployment of the Global Capstone Project embedded in the MSIT-funded convergence initiative Development of the HST-K joint undergraduate major Award of MOHW-funded 5-year national R&D grant for medical AI education and talent development (2025–2029)

## Design principles: lessons from the institutional case

The phased rollout of convergence education at SNU Medicine from 2020 to 2025 highlights several design principles that can guide future efforts in developing medical AI curricula. These principles extend beyond a single institutional case and provide a framework for theorizing the structure, governance, and transferability of interdisciplinary education in the context of rapidly advancing digital technologies.

Building on this institutional trajectory, we identified four interconnected design principles: (1) modularity, which enables flexible integration of convergence content across degree programs; (2) transdisciplinary alignment, which fosters deep collaboration among medicine, engineering, and data science; (3) infrastructure–curriculum coupling, which directly links research platforms with educational programs to maintain continuity and real-world relevance; and (4) policy embeddedness, which connects institutional reforms with national digital health strategies and funding frameworks. Together, these principles create a transferable framework for developing sustainable and scalable medical AI education. Table 2 presents each design principle alongside its institutional implementation and implications for transferability, directly linking the case analysis to broader applicability.

**Table 2:** Design principles for institutionalizing medical AI education

Design principle	Definition & purpose	Implementation at SNU medicine	Implications for transferability
Modularity and Cross-Credit Integration	Offering adaptable, stackable educational modules that integrate into current curricula	Microdegree, integrated BA–MS, elective intensives	Minimal disruption; easily adaptable to various institutions with curriculum restrictions
Transdisciplinary Alignment	Deep collaboration across disciplines beyond co-teaching	Joint course development with faculty from medicine, engineering, data science, and industry	Promotes shared ownership; develops new professional identities
Infrastructure–Curriculum Coupling	Connecting research centers with educational programs	MBRC and SNU AI.MED function as both research and teaching platforms	Enhances continuity, realism, and scalability
Policy Embeddedness	Aligning education with national digital strategies and funding sources	Multi-year national grants (e.g., MOHW, MSIT) aligned with institutional programs and international initiatives	Ensures legitimacy and long-term sustainability

### Modularity and cross-credit integration

Modularity allows educational content to be delivered in separate, flexible units that can be added without completely changing existing curricula. At SNU Medicine, this idea made it possible to introduce AI-related courses gradually across current programs through credit-bearing modules, short-term intensives, and microdegree certificates.

For example, the Medical AI Microdegree Program, offered in collaboration with the College of Engineering and Graduate School of Data Science, was designed to operate independently or alongside existing graduate programs. Similarly, the integrated bachelor’s–master’s pathway allowed medical students to participate in data science research while completing their core MD training, with cross-campus credits officially recognized through joint governance structures.

This approach reduced institutional resistance by minimizing disruption to core curricula and encouraged participation from students across disciplines. It also supported a long-term strategy for scalable, multi-entry-point convergence education that can adapt to future needs.

### Transdisciplinary alignment

Transdisciplinary alignment requires more than just interdisciplinary exposure. It involves shared ownership of curricula, co-creating learning objectives, and integrated instruction across clinical, technical, and institutional boundaries. At SNU Medicine, convergence education was not limited to co-teaching arrangements or elective offerings. Instead, it was rooted in deeply collaborative models that brought together faculty from the medical school, university hospitals, and high-tech industry.

One illustrative example is the course “Deep Learning for Healthcare,” jointly developed by SNU Medicine, clinical faculty from university hospitals, and AI researchers from a leading tech company. Instead of relying on guest lectures, the course was co-designed and co-taught, with unified content that combined clinical relevance, engineering fundamentals, and real-world algorithmic applications. This approach showed an integrated model where institutional roles and disciplinary boundaries were intentionally crossed to create a cohesive learning experience.

Another example is the pre-medical course “Healthcare and Data Science,” introduced in 2023. A curriculum development committee—composed of professors from SNU Medicine and clinical faculty from university hospitals—collaboratively set the course goals, chose core competencies, and developed the syllabus. This ensured that early-stage students gained exposure to computational thinking, ethical data practices, and informatics rooted in both academic theory and clinical practice.

These initiatives demonstrate a shift from multidisciplinary coordination to genuine transdisciplinary integration. Educators from different fields collaboratively took responsibility for designing, delivering, and assessing educational content, encouraging a culture of shared ownership. As a result, students were able to develop professional identities that go beyond traditional disciplinary boundaries and prepare for complex roles in AI-enabled healthcare systems.

### Infrastructure–curriculum coupling

One of the most distinctive features of the SNU Medicine model was how it combined research infrastructure with educational design. Instead of seeing research centers as separate from teaching, the college used its MBRC and SNU AI.MED as platforms for both innovation and education.

The MBRC provided access to unstructured clinical data—such as surgical videos, biosignals, and clinical audio—which was directly incorporated into project-based learning modules in both undergraduate and graduate courses. Medical and engineering students participated in joint data challenges, hackathons, and co-op programs based on real-world hospital datasets, under the joint supervision of clinician-researchers and data scientists. This not only enhanced students’ technical skills but also deepened their understanding of actual clinical workflows.

SNU AI.MED was more than just a course oversight committee. It acted as an academic governance body that coordinated interdisciplinary faculty teams, ensured quality control across programs, and collaborated with national funding agencies to align educational design with digital health policy priorities. This dual role—both internally focused on curriculum design and externally focused on policy translation—was essential for maintaining the program’s sustainability beyond the pilot phase.

By integrating convergence education into these infrastructure platforms, SNU Medicine addressed a common limitation of curriculum reform efforts: the disconnect between teaching and institutional resources. The result was a model that provided continuity, real-world relevance, and strategic scalability, grounded in both academic rigor and national innovation priorities.

### Policy embeddedness

Policy embeddedness involves integrating institutional education models with national priorities, funding mechanisms, and long-term innovation objectives. At SNU Medicine, this integration was crucial for ensuring sustainability, scalability, and institutional legitimacy for convergence education.

From its beginning, the program was developed closely with Korea’s Digital New Deal, MOHW’s AI talent development initiatives, and national R&D policies focused on digital health transformation. For example, the launch of SNU AI.MED was made possible by a multi-year government grant aimed at promoting cross-disciplinary education and research in AI and medicine. This grant not only provided funding but also established evaluative criteria that encouraged institutional integration and collaboration across departments and campuses.

These national programs significantly influenced the design and structure of convergence education at SNU Medicine. The Medical AI Microdegree, for instance, was created in line with government initiatives that focused on modular, industry-relevant credentialing frameworks to quickly enhance AI skills in the healthcare workforce. Instead of passively following policy incentives, the institution strategically used them to promote its internal vision and gain structural support for lasting educational reform.

SNU AI.MED served both as a curriculum platform and strategic interface—linking academic initiatives with public funders, industry partners, and evolving accreditation standards. This dual role allowed the institution to secure multi-year funding, build strong cross-campus governance, and expand offerings beyond what internal resources alone could support. More importantly, it ensured that convergence education was not seen as a minor experiment but as a nationally important institutional priority integrated into Korea’s broader innovation agenda.

## Policy and global implications

The institutionalization of convergence education at SNU Medicine offers policy-relevant insights for other medical schools and public universities aiming to incorporate AI into their educational missions. While some aspects of the model are specific to its context—shaped by Korea’s national innovation agenda and institutional governance structures—several features have broader relevance. This section emphasizes key policy implications and potential pathways for transfer.

These challenges were not limited to institutional governance. At the faculty level, workload intensified as instructors balanced research productivity with new teaching responsibilities, while also adapting to evolving government policy benchmarks. At the student level, many medical students entered the program with little or no prior programming experience. To address this gap, the curriculum was adjusted by adding scaffolded introductory modules, peer mentoring systems, and project-based assignments that allowed students to engage meaningfully regardless of prior technical background.

### Recognizing what is Scalable—and what is not

One main challenge in interpreting the SNU Medicine model is identifying which components are universally applicable and which are closely connected to local policy conditions. For example, modular curricula, cross-credit systems, and interdisciplinary co-teaching arrangements can be adopted across different institutional settings, as long as institutions have sufficient administrative coordination and curricular flexibility. These elements are design choices rather than essential policy requirements.

In contrast, components like centralized funding for organizational platforms (e.g., SNU AI.MED) and ongoing support from ministries (such as Health and Welfare or Education) rely heavily on Korea’s centralized higher education and R&D policy environment. Institutions in other countries may lack comparable national infrastructure or incentives to align education with strategic innovation goals. In such contexts, the SNU Medicine model functions less as a directly replicable template and more as a principle-based framework for local adaptation. In this regard, even in contexts without centralized funding, institutions can still adopt a governance-driven convergence platform by leveraging existing university–hospital partnerships and aligning modular microdegrees with specific industry needs (e.g., local clinical AI vendors), thereby securing legitimacy and resources at a sectoral rather than national level.

### Aligning convergence education with National innovation policy

The SNU Medicine experience shows how convergence education can be incorporated into a larger national innovation strategy. From the Digital New Deal to the Government Grant Program for Education and Research in Medical AI, Korea’s policy environment offered clear signals, funding sources, and performance benchmarks that helped establish convergence as a key institutional focus. This enabled convergence efforts to connect multiple policy areas—tying educational reform to broader goals in healthcare innovation and industrial development.

Such alignment might be less automatic in countries with fragmented policy systems or decentralized education governance. However, the core principle remains the same: for convergence education to grow and last, it must be clear to policymakers, funders, and accreditation bodies. Programs that directly meet national workforce needs—especially in digital health—are more likely to gain legitimacy and sustain long-term growth. In contexts lacking centralized policy or funding support, convergence education can still be advanced through incremental yet sustainable strategies. Embedding AI literacy into existing medical curricula—rather than building new programs from scratch—ensures curricular continuity. Project-based learning, anchored in locally available datasets or case studies, can provide meaningful training without requiring heavy infrastructure. Professional societies and accreditation bodies can play a legitimizing role by setting competency standards in the absence of national mandates. Finally, institutions can adopt a stepwise approach, beginning with small-scale elective modules and scaling up as faculty capacity and student demand grow.

When compared with international benchmarks, the uniqueness of the SNU Medicine model becomes clearer. The Harvard–MIT Health Sciences and Technology (HST) program exemplifies an elite, dual-degree pathway that immerses a small group in high-intensity interdisciplinary research, whereas SNU Medicine has integrated convergence across its entire institutional structure, from undergraduate to postgraduate levels. At Imperial College London, modular digital health curricula, such as the Health Data Analytics and Machine Learning Master’s program, offer advanced specialization but remain specific to each program. In contrast, SNU’s modular microdegrees and cross-credit integration are institution-wide and managed by the institution. Georgia Tech and Emory’s joint biomedical engineering curriculum emphasizes engineering-led AI literacy with health applications. At the same time, SNU Medicine grounds AI training in core medical education and aligns it directly with national policy priorities. These differences suggest that although SNU benefited from Korea’s centralized policy environment, its design principles—particularly modularity and infrastructure–curriculum coupling—can be adapted by institutions in decentralized or resource-limited settings through local governance mechanisms and sectoral partnerships.

### Implications for other public medical institutions

Public medical institutions worldwide face unique challenges in adopting new educational models. They often work within strict curricula, have limited financial and faculty resources, and must balance academic objectives with public service duties. However, as digital transformation reshapes healthcare, the need for convergence education in these settings becomes more urgent.

Focusing on modularity, flexible credentialing, and cross-sector governance provides a practical framework for building scalable, publicly responsible models of medical AI education. By integrating convergence programs into university–hospital systems, public institutions can utilize their clinical settings not only for structured learning but also as dynamic laboratories for applied research, innovation, and ethical assessment.

## Conclusion: rethinking medical education in the AI era

As AI continues to transform medicine, few public institutions have documented how long-term, strategic curriculum reform can be effectively implemented. The case of SNU Medicine provides one such example. Between 2020 and 2025, the institution progressed through three interlinked stages: establishing foundational elements, enhancing its structural capacity, and developing national and international collaborations. These stages collectively enabled the creation of a convergence education model that integrates AI, data science, and clinical expertise into medical training.

This phased approach reveals an important insight: effectively integrating AI into medical education requires more than just changing the curriculum. It calls for reorganization at several levels, including institutional structures, faculty development, credit systems, and policy alignment. At SNU Medicine, convergence education did not result from a single change, but rather from the layering of modular programs, fostering transdisciplinary collaboration, and integrating infrastructure into the curricula. By connecting education with research platforms, aligning teaching with national strategies, and encouraging collaboration across departments and sectors, the institution created a model that many still aim to emulate.

Instead of serving as a one-size-fits-all solution, the SNU Medicine model offers a flexible framework based on four key principles: modularity, which allows gradual integration without a complete curriculum overhaul; transdisciplinary alignment, which encourages cross-field collaboration; infrastructure–curriculum coupling, which integrates education into research and institutional platforms; and policy embeddedness, which links educational innovation to national strategic goals. These principles provide a transferable foundation that can be adapted to various institutional and policy environments.

Looking ahead, the challenge is not only teaching AI tools to medical students but also preparing them to shape the development, use, and governance of AI in healthcare. As algorithmic systems become vital to clinical practice, public health, and biomedical discovery, the boundaries between user and creator—clinician and computationalist—will continue to blur. Medical education must evolve by fostering AI-literate physicians who can interpret, critique, and co-develop the technologies they use.

While the SNU Medicine case does not claim to provide a universal blueprint, it shows how convergence education—when built into the structure and strategically coordinated—can serve as a practical model for rethinking medical training in an AI-driven healthcare future.
